# The diagnostic and prognostic value of the miR-17-92 cluster in hepatocellular carcinoma: A meta-analysis

**DOI:** 10.3389/fgene.2022.927079

**Published:** 2022-09-02

**Authors:** Fang Lu, Xianghong Zhao, Zhongqiu Zhang, Mengqiu Xiong, Ying Wang, Yalan Sun, Bangshun He, Junrong Zhu

**Affiliations:** ^1^ School of Basic Medicine and Clinical Pharmacy, China Pharmaceutical University, Nanjing, China; ^2^ Department of Pharmacy, Nanjing First Hospital, China Pharmaceutical University, Nanjing, China; ^3^ Department of Laboratory Medicine, Nanjing First Hospital, Nanjing Medical University, Nanjing, China

**Keywords:** miR-17-92 cluster, hepatocellular carcinoma, diagnostic value, prognostic value, meta-analysis

## Abstract

Previous studies demonstrated that microRNAs (miRNAs) could serve as biomarkers in various cancers. This meta-analysis aimed to determine the roles of a miR-17-92 cluster in hepatocellular carcinoma (HCC). Here, eligible included studies were searched through PubMed, Embase, and Wan Fang databases up to 1st February 2022. Relevant data were extracted from each eligible study to evaluate the relationship between miRNA-17-92 cluster miRNA expression and the diagnosis and prognosis of HCC. Finally, a total of 21 studies were pooled and included in the meta-analysis, of which four articles were used for diagnostic meta-analysis and eight articles were used for prognostic meta-analysis. The pooled sensitivity, specificity, and diagnostic odds ratios (DOR) of the miR17-92 cluster for diagnosis of HCC were 0.75 [95% confidence interval (CI): 0.64–0.83], 0.73 (95% CI: 0.65–0.79), and 7.87 (95% CI: 5.36–11.54), respectively. Also, the area under the curve (AUC) for the miR-17-92 cluster when diagnosing HCC was 0.79 (95% CI: 0.76–0.83). For prognostic analysis, hazard ratios (HRs) with 95% CIs were extracted from the included studies and pooled HRs were determined to assess the associations. Patients with increased expression of miR17-92 cluster miRNA were associated with poor overall survival (OS) and recurrence-free survival (RFS) (HR=1.86, 95% CI: 1.04–3.33; HR = 4.18, 95% CI: 3.02–5.77, respectively), but not progression-free survival (PFS) (HR = 0.43, 95% CI: 0.25–0.73), while no association of the miR-17-92 cluster high-expression was detected with disease-free survival (DFS) (HR: 0.95, 95% CI: 0.21–4.34). In short, current pieces of evidence suggested that the miR-17-92 cluster may serve as a novel diagnostic and prognostic biomarker for HCC. However, given the limited study number, larger-size, multi-center, and higher-quality studies are indispensable in the future.

## Introduction

Hepatocellular carcinoma (HCC) is one of the most malignant tumors, accounting for approximately 90% of primary liver cancers (Llovet et al., 2021). Worldwide, it ranks sixth in incidence and fourth in cancer death-related causes (Villanueva, 2019). As a country with a relatively high incidence of HCC, China accounts for more than 50% of the world's annual new cases (4,66,000) and deaths (approximately 4,22,000) each year (Chen et al., 2016; Gordan et al., 2020), which are mainly due to the highly complex heterogeneity and multiple etiologies of HCC, including cirrhosis, hepatitis B virus (HBV), hepatitis C virus (HCV) infection, and nonalcoholic steatohepatitis (NASH) (Estes et al., 2018; Zhang et al., 2022). Moreover, aflatoxin-contaminated foodstuffs, heavy alcohol intake, obesity, smoking, type 2 diabetes, and other related cofactors contribute to the pathogenesis of HCC (Llovet et al., 2016).

Currently, alpha-fetoprotein (AFP), a carcinoembryonic glycoprotein, is used alone or in combination with ultrasound and other imaging modalities as a tumor marker for HCC, whereas its utility is limited due to low sensitivity and specificity, as well as differences between different measurement methods, which were attributed to patient characteristics, study design, and AFP cutoff values (Gupta et al., 2003). The traditional treatments such as surgical resection, liver transplantation, local minimally invasive, precision radiotherapy, radical treatment, systemic treatment (targeted therapy and immunotherapy), optimal treatment, and palliative treatment were mainly selected for HCC patients. However, due to the high recurrence rate and high metastasis rate of HCC, the prognosis of HCC has always been unsatisfactory. The 5-year survival rate of patients with early HCC is as high as 75%, while the 1-year survival rate of patients diagnosed with generalized cancer is less than 10% (El-Serag et al., 2008; El-Serag, 2011). The HCC has a serious impact on human health and quality of life around the world. Despite reasonable and well-established treatment options, the prognosis of patients is also very poor because HCC is often diagnosed at a rapidly progressive and advanced stage. Hence, it is urgent to further understand the mechanism underlining the pathological progression of HCC and find new markers for early diagnosis and treatment prognosis.

MicroRNAs (miRNAs) are a type of short, single-stranded non-coding RNAs with 19–25 nucleotides regulating the gene expression mainly by targeting the 3′-UTR of mRNAs, posttranscriptional effects (O’Brien et al., 2018). Therefore, they are involved in regulating numerous biological events such as apoptosis, cell cycle, proliferation, and invasion, playing key roles in tumorigenesis and malignant development (Gebert and MacRae, 2019). Accumulating studies have demonstrated that abnormal expression of miRNAs could serve as the biomarker for early diagnosis and treatment of HCC. For example, combining serum miR497 and miR-1246 together for HCC diagnosis, the sensitivity and specificity reach 94.0% and 70.0%, respectively, which increased the accuracy of HCC diagnosis (Chen et al., 2021). Huang et al. (2021) found that the expression of miR-125b-2-3p was downregulated in HCC tissues compared with non-tumor tissues, and the lower expression of miR-125b-2-3p indicated poor progression and prognosis in HCC. El-Mezayen et al. (2021) found that miR-25 modulated an oncogenic function by regulating the Ubiquitin Ligase Fbxw7 in HCC.

The miR-17-92 is a typical highly conserved miRNA cluster, which is found in human chromosome 13 open-reading frame 25 (C13orf25) (Fang et al., 2017). The cluster encodes six mature miRNAs, namely, miR-17, miR-18a, miR-19a, miR-19b, miR-20a, and miR-92a (Ota et al., 2004; Concepcion et al., 2012; Zhang et al., 2018). Recently, multiple studies have shown that members of the miRNA-17-92 cluster are specifically expressed in types of cancer, suggesting that the miR-17-92 cluster may serve as a new biomarker for cancer diagnosis and treatment. For example, miR-92a is closely associated with colorectal cancer lymphoma metastasis, suggesting that miR-92a may be a potential marker of colorectal cancer (Zhou et al., 2013); in a meta-analysis on the diagnostic value of serum miRNA in gastric cancer, it was indicated that the area under the curve (AUC) for the combination of miR-19a and miR-92a was the highest at 0.850, with a sensitivity of 91.3% and a specificity of 61.0% (Liu et al., 2018). However, evidence for the diagnostic and prognostic role of miR-17-92 cluster expression in HCC was still lacking. Therefore, this study aims to explore the diagnostic and prognostic value of the miR-17-92 cluster in HCC by investigating expression levels of this miRNA in HCC patients. The findings of this study might provide a potential reference for clinicians

## Materials and methods

### Literature search

A comprehensive literature search was conducted in PubMed, Embase, and Wan Fang, with an update on 1st February 2022 to obtain potentially eligible studies. The search was conducted with the keywords (hepatocellular carcinoma OR HCC) AND (miR-17 OR microRNA-17 OR miR-18a OR microRNA-18a OR miR-19a OR microRNA-19a OR miR-19b OR microRNA-19b OR miR-92a OR microRNA-92a OR miR-20a OR microRNA-20a) AND (survival OR prognosis OR outcomes OR diagnosis). The searches were limited to human studies and articles in English. Additionally, other relevant articles were also obtained by manually screening the reference lists.

### Inclusion and exclusion criteria

Inclusion criteria for the studies were as follows: 1) the study’s patients had been diagnosed with HCC (any stage or histology) and tumor samples are naïve or associated with HBV/HCV; 2) clinical study about the association of expression of the miR-17-92 cluster with HCC diagnostic or prognostic value; 3) studies investigating the expression in whole blood, plasma or serum or tissues or Formalin-Fixed and Paraffin-Embedded (FFPE) of the miR-17-92 cluster in HCC patients and healthy controls; 4) the sensitivity and specificity are reported to provide enough information to construct a 2 × 2 contingency table, which includes true positive, false positive, false negative, and true negative; 5) relevant available data of the hazard ratios (HRs) and their corresponding 95% confidence intervals (CIs) to evaluate its associations could be obtained; and 6) patient prognostic outcomes including overall survival (OS), recurrence-free survival (RFS), disease-free survival (DFS), and progression-free survival (PFS).

Studies were excluded according to these exclusion criteria as follows: 1) duplicate publications; 2) other types of articles, such as meeting minutes, abstracts, comments, meta-analysis, patents, case reports, and letters; 3) insufficient data, unable to calculate true and false positive and negative information and unable to calculate HRs and 95% CIs; and 4) diagnostic or prognostic data from the TCGA data set.

### Data extraction and quality assessment

The studies of diagnostic value extracted the following data: first author’s name, publication year, study country, sample size, sample type, sample stage, microRNA, test method, and cut-off value; data are extracted by designing a table that includes sensitivity, specificity, number of true positives, number of false positives, number of false negatives, and number of true negatives. The study of prognostic value extracted the following information: first author’s name, publication year, study country, sample size, sample type, sample stage, microRNA, test method, cut-off value, outcome indicator, and HRs along with their corresponding 95% CIs.

Two researchers (Fang Lu and Xianghong Zhao) independently assessed whether each included study met the quality standards. Then, another researcher (Zhongqiu Zhang) reevaluated and made a unified conclusion if there was a discrepancy between the first two researchers. The quality assessment of each included diagnosis-related study was performed with the Quality Assessment of Diagnostic Accuracy Studies 2 (QUADAS 2) (Whiting et al., 2003), which is considered reliable for the quality assessment of test accuracy studies. The quality of involved prognosis-related studies was evaluated with the Newcastle–Ottawa Scale (NOS) (Lo et al., 2014), which is the tool most commonly used to assess the quality of non-randomized research (Roysri et al., 2014).

### Statistical analysis

For diagnostic accuracy studies, the sensitivity, specificity, positive likelihood ratio (PLR), negative likelihood ratio (NLR), and corresponding 95% CIs from included studies were pooled to preliminarily assess the diagnostic value of the miR-17-92 cluster in HCC. Then, based on the original data, the summary receiver operating characteristic (SROC) curve was drawn, and the AUC was calculated to comprehensively determine the diagnostic accuracy of the miR-17-92 cluster. To assess the heterogeneity across studies, the Q-statistic and *I* ^2^ statistics were utilized. The *I* ^2^ value typically fluctuates within a range of 0 (unobserved heterogeneity) to 100% (maximum heterogeneity). *P* value < 0.05 or *I* ^2^ > 50% was recognized statistically significant (Raponi et al., 2009). If the studies were proved to be heterogeneous, the random-effects model would be utilized for further analysis. Subsequently, subgroups were analyzed to find potential sources of heterogeneity. Finally, the publication bias of all the included diagnostic accuracy studies was assessed by Deek’s funnel plots Deeks et al. (2005) (significant at *P* < 0.05).

For the prognostic meta-analysis, the HRs and 95% CIs extracted from the eligible studies were combined to elucidate the relationship between the expression of miR-17-92 of cluster members and the survival results of HCC. *I* ^2^ statistics were applied to perform the heterogeneity of the pooled results (Higgins et al., 2003). The heterogeneity of the combined HRs would be considered acceptable if *I* ^2^ was <50%. In addition, the publication bias of all the included prognostic studies was evaluated by funnel plots and by Begg’s and Egger’s tests. *P* < 0.05 suggests the existence of publication bias in studies (Begg and Berlin, 1989). All abovementioned statistical calculations were carried out with STATA Statistical Software Version 12.0 (Stata Corp, College Station, TX, SA) and Excel software 2019.

## Results

### Summary of included study characteristics

**FIGURE 1:**
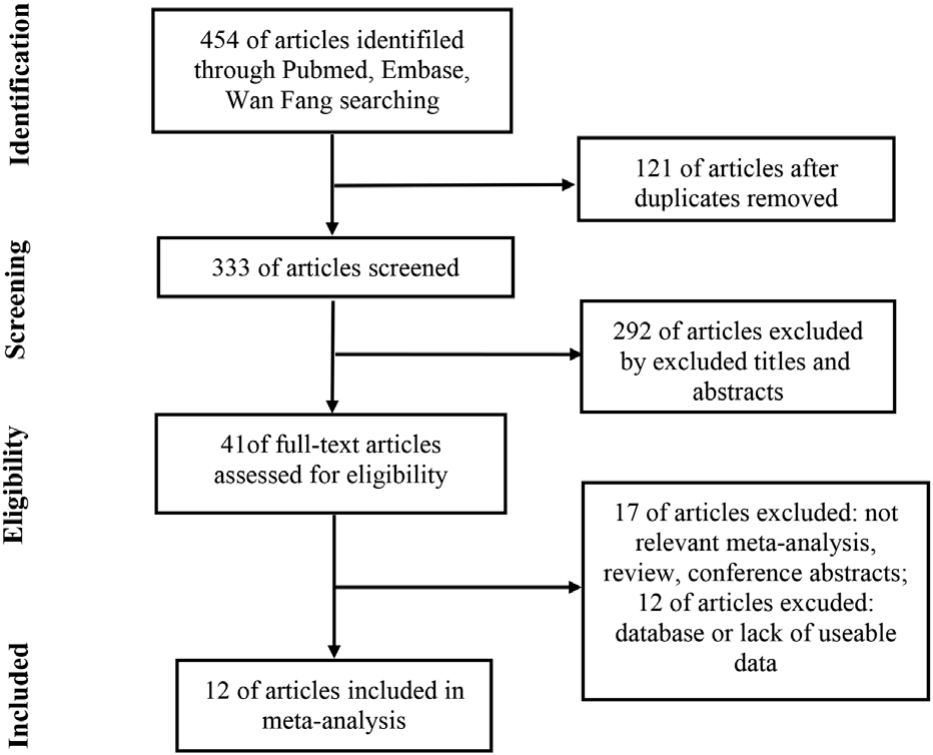
Flow chart of the article selection process.

The detailed article retrieval process is shown in Figure 1. Initially, a total of 454 articles were retrieved by the keywords, of which 121 duplicate articles were removed by checking article titles. After screen titles and abstracts of 292 articles were further removed, a total of 41 articles were all downloaded to obtain valid information individually. After reading the full text, 17 articles were removed due to irrelevant meta-analyses, reviews, and conference abstracts. Meanwhile, 12 articles were excluded due to the database or lack of useable diagnostic or prognostic data. Finally, 12 articles that met all the inclusion criteria were included in the current study. All enrolled eligible articles were published from 2012 to 2020, accumulating 1039 HCC patients and 349 case-control subjects.

### The results of the diagnostic meta-analysis of hepatocellular carcinoma

Six studies (Li et al., 2012; Motawi et al., 2015; Wen et al., 2015; FathyElmougy et al., 2019) reported the role of the miR-17-92 cluster as a biomarker in HCC diagnosis, including 320 HCC patients and 349 control subjects. The characteristics and methods related to the diagnostic accuracy of the included studies are shown in Table 1. Of which, two ethnic groups were analyzed, three studies from Asians and the remaining three from Africans. All studies were performed with serum or plasma. Moreover, all included studies used real-time quantitative real-time polymerase chain reaction (qRT-PCR) to detect miR-17-92 miRNA expression.

**TABLE 1:** Main characteristics of the eligible studies for diagnostic meta-analysis.

First author	Year	Country	Sample source	Sample range	Patient case/control	microRNA	Test method	SEN (%)	SPE (%)	Tp	FP	FN	TN	Cut off	QUADAS-2
Yang Wen	2015	China	Plasma	I-IV	67/82	miR-20a	qRT-PCR	86.6	57.3	58	35	9	47	2.555 × 10^–3^	5
Yang Wen	2015	China	Plasma	NR	67/82	miR-92a	qRT-PCR	76.1	68.3	51	26	16	56	5.573 × 10^–3^	5
Lihua Li	2012	China	serum	NR	101/60	miR-18a	qRT-PCR	86.1	75	87	15	14	45	1.765	6
Tarek K. Motawi	2015	Egypt	serum	NR	112/42	miR-19a	qRT-PCR	60.7	89.2	68	5	44	37	<0.625	7
Tarek K. Motawi	2015	Egypt	serum	NR	112/125	miR-19a	qRT-PCR	92.9	75.5	68	31	44	94	<1.58	7
Fatma A. Fathy.Elmougy	2019	Egypt	serum	NR	40/40	miR-19a	qRT-PCR	70	77.5	28	12	9	31	≥0.65	5

NR, not report; qRT-PCR, quantitative reverse transcription-polymerase chain reaction; SEN, sensitivity; SPE, specificity; TP, true positive; FP, false positive; FN, false negative; TN, true negative; QUADAS-2, quality assessment of diagnostic accuracy studies 2.

**FIGURE 2:**
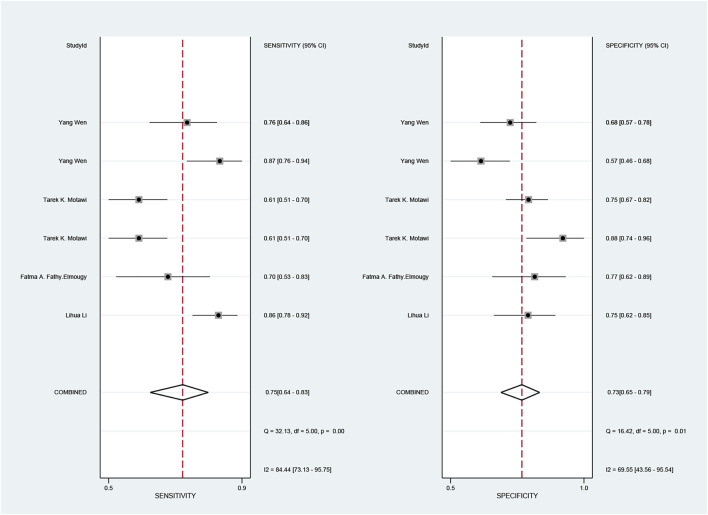
Forest plot of sensitivity and specificity for the miR-17–92 cluster in the diagnosis of hepatocellular carcinoma.

**FIGURE 3:**
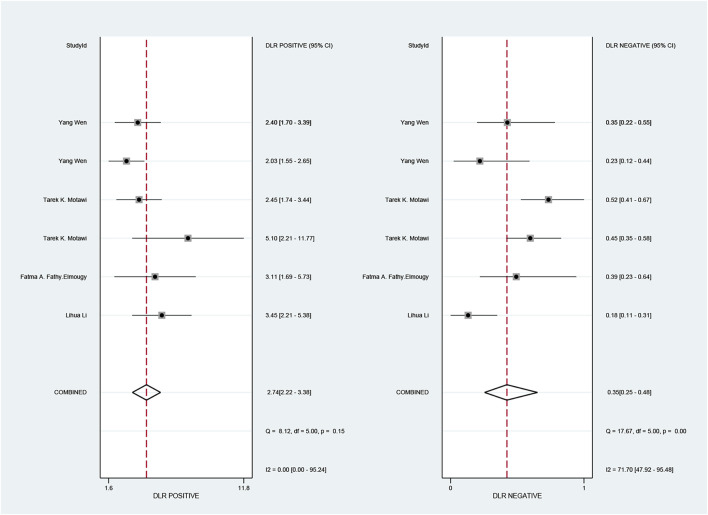
Forest plot of positive likelihood ratios (PLRs) and negative likelihood ratios (NLRs) for the miR-17–92 cluster in the diagnosis of hepatocellular carcinoma.

**FIGURE 4:**
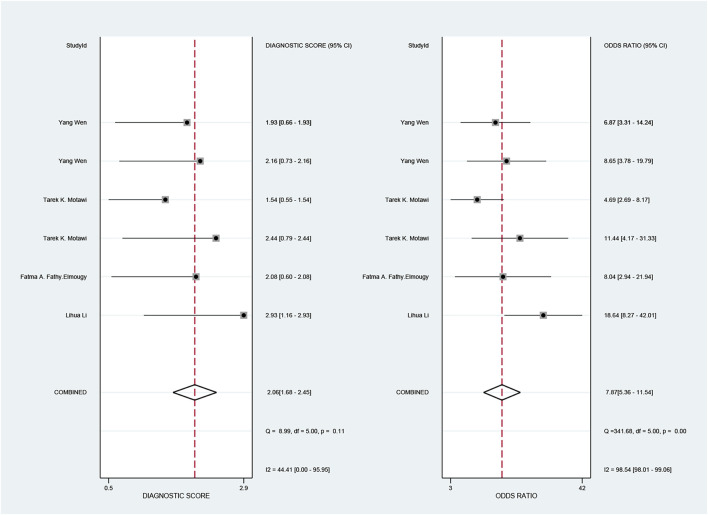
Forest plot of the diagnostic odds ratio (DOR) for the miR-17–92 cluster in the diagnosis of hepatocellular carcinoma.

**FIGURE 5:**
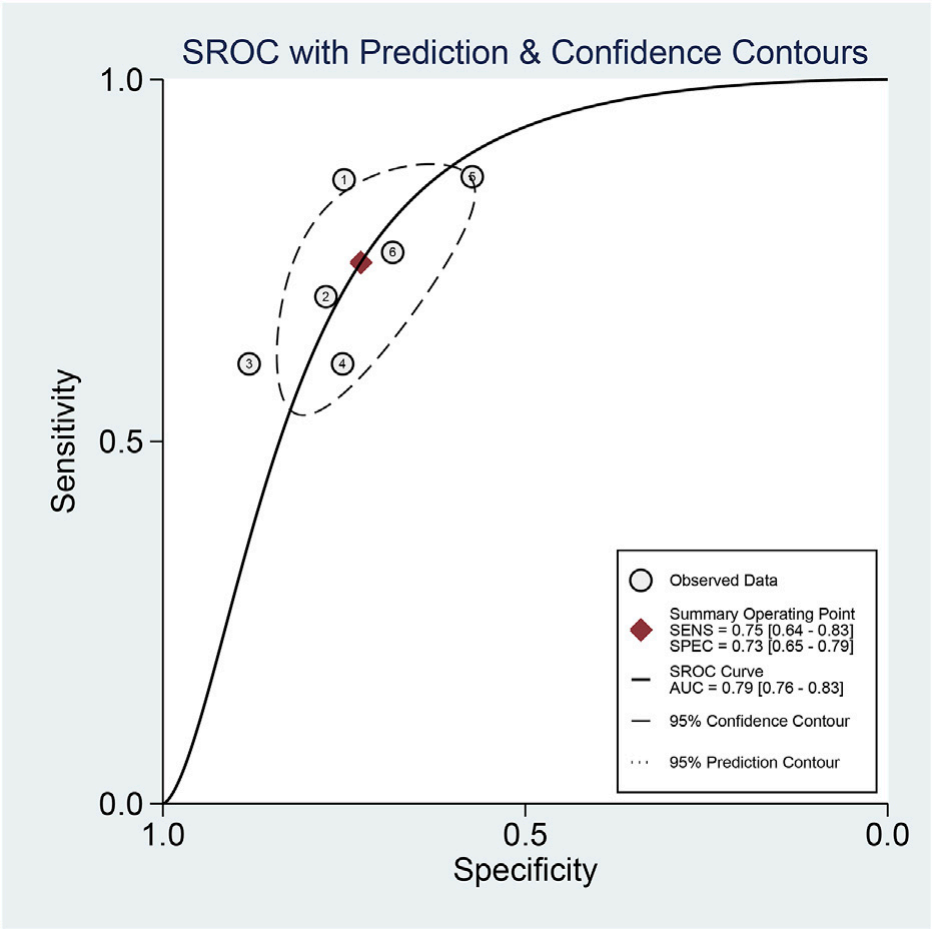
Summary receiver operating characteristic curve (SROC) for the miR-17–92 cluster in the diagnosis of hepatocellular carcinoma.

The pooled sensitivity and specificity of the diagnostic value were 0.75 (95% CI: 0.64–0.83) and 0.73 (95% CI: 0.65–0.79), respectively, as shown in Figure 2. Meanwhile, the PLR and NLR were 2.74 (95% CI: 2.22–3.38) and 0.35 (95% CI: 0.25–0.48), respectively, which means that the probability of miR-17-92 positive in HCC patients was 2.74 times higher than that in controls, while HCC patients had miR-17-92 which was 0.35 times more likely to be negative than that of non-patients, shown in Figure 3. In addition, the pooled Diagnostic Odds Ratio (DOR) was 7.87 (95% CI: 5.36–11.54), as shown in Figure 4, and the area under the SROC (AUC) was 0.79 (95% CI: 0.76–0.83), suggesting that the miR-17-92 cluster has an acceptable diagnostic value, shown in Figure 5.

**FIGURE 6:**
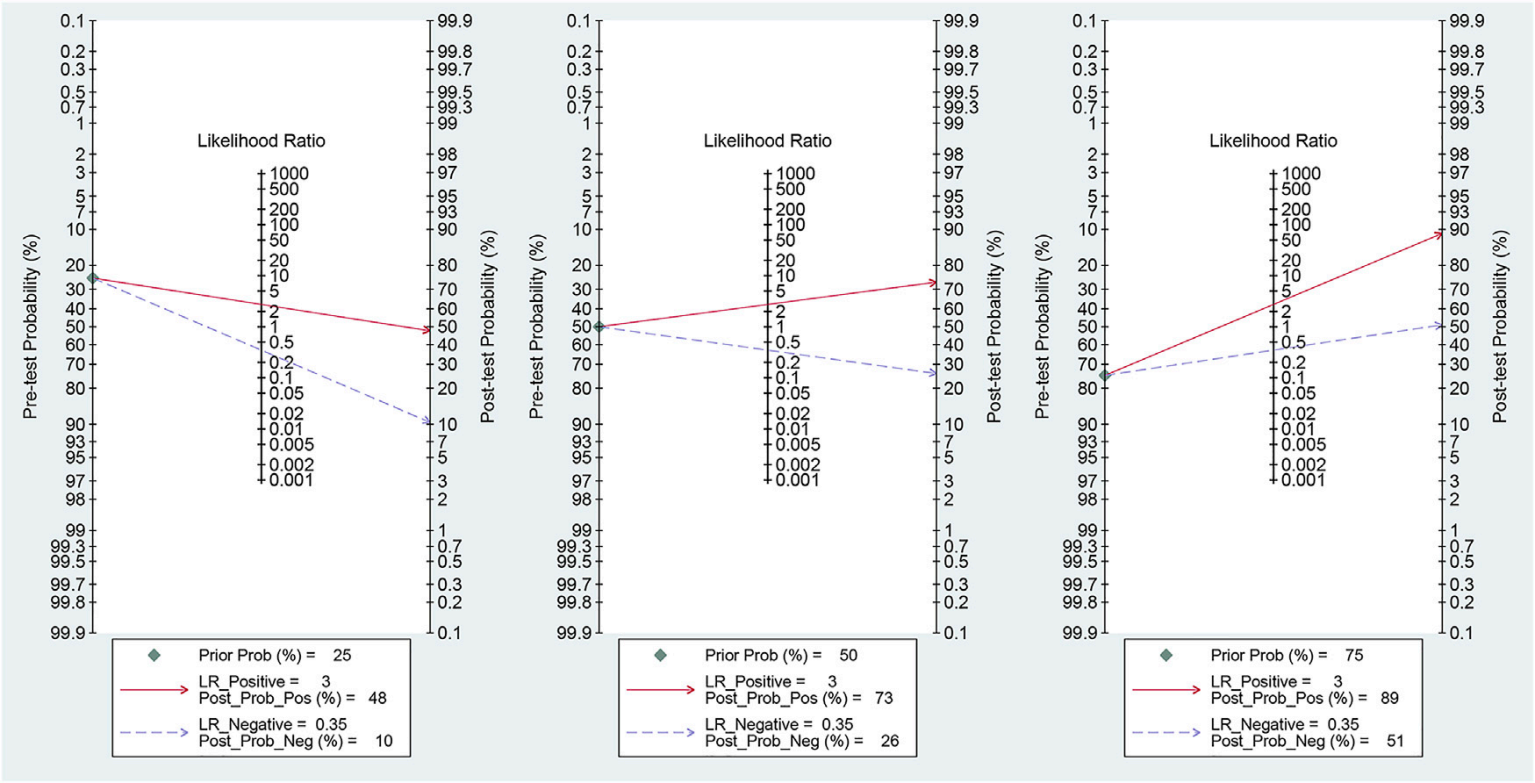
Fagan plots for the miR-17–92 cluster with 25, 50, and 75% pre-test probability of diagnosing hepatocellular carcinoma.

**FIGURE 7:**
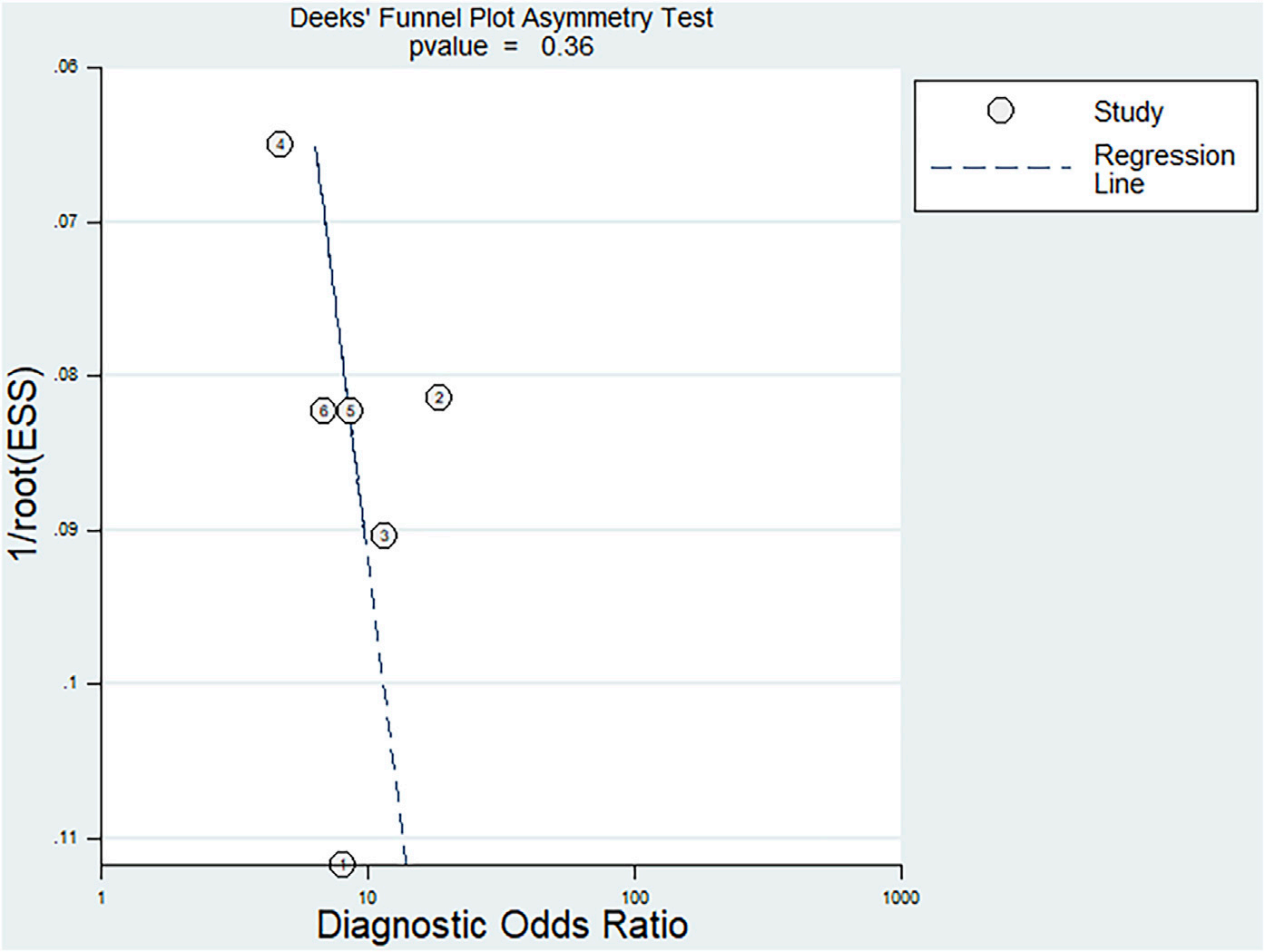
Deek’s funnel plot asymmetry test for assessing publication bias.

Fagan’s nomogram was applied for assessing the clinical utility of the index test. We used likelihood ratios to simulate three clinical scenarios by implementing different pretest probabilities, with 25%, 50%, and 75% representing relatively low, moderate, and relatively high clinical suspicion, respectively. We used these likelihood ratios to evaluate post-test probabilities and plot the Fagan nomogram, as shown in Figure 6. When the pretest probability was 25%, the posttest probability positive (PPP) and the posttest probability negative (PPN) was 48% and 10%, respectively. When the pre-test probability is 50%, the PPP and PPN are 73% and 26%, respectively; when the pre-test probability is 75%, the PPP and PPN are 89% and 51%, respectively. Taken together, the miR-17-92 cluster had relatively acceptable accuracy for the identification of HCC patients. Deek’s funnel plot showed that *P*-value across the studies was 0.36, indicating that there is almost no publication bias in diagnostic meta-analysis, as shown in Figure 7.

**FIGURE 8:**
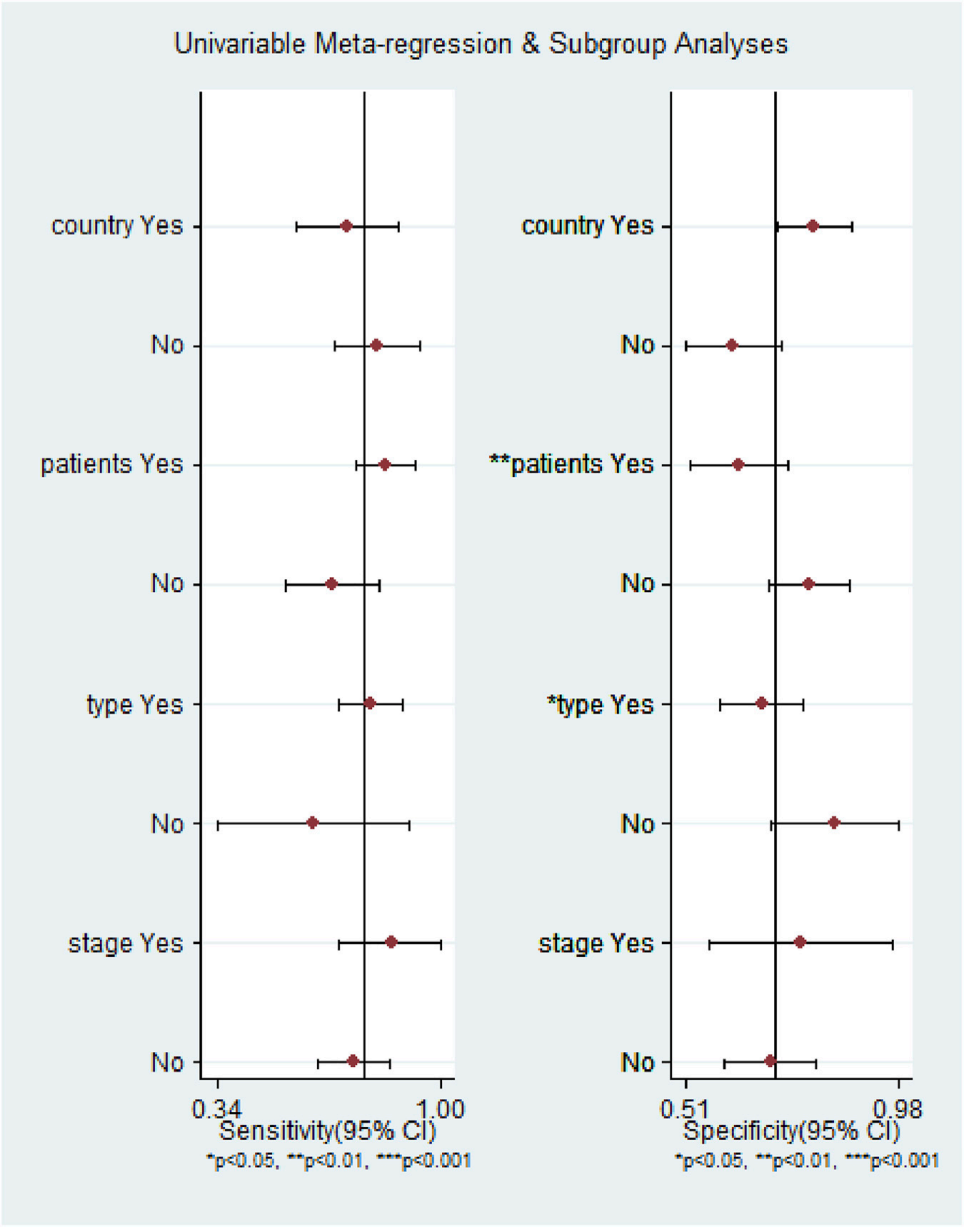
Meta-regression analysis to detect the source of heterogeneity for the miR-17–92 cluster in the diagnosis of hepatocellular carcinoma.

The ROC curve was a nontypical “shoulder–arm” appearance, so there was no significant threshold effect in the current meta-analysis, as shown in Figure 5. Moreover, the Spearman correlation coefficient between the log of sensitivity and the log of specificity was −0.95 (*p* = 0.90), also showing no significant threshold effect existing. According to the results of diagnostic accuracy analysis, significant heterogeneity was found across studies of sensitivity (*I* ^2^ = 84.44%, *p* = 0.00) and specificity (*I* ^2^ = 69.55%, *p* = 0.01), which suggested the significant heterogeneity caused by the non-threshold effect has existed among these studies. Hence, a meta-regression was used to distinguish the potential origins of heterogeneity between studies by exploring research characteristics, such as country, patients, sample type, and sample stage, and the results showed that heterogeneity was mainly derived from patients (*P* < 0.01) and sample type (*P* < 0.05), as shown in Figure 8.

The subgroup analysis results based on sample type, sample size, country, and tumor stage are summarized in Table 2. Stratified analysis by sample type showed that the sensitivity, specificity, and AUC of miR-17-92 cluster miRNA expression in serum were 0.70 (95% CI: 0.58–0.83), 0.77(0.72–0.82), and 0.79, respectively. However, since only two studies were included in the miR-17-92 cluster miRNA expression in plasma, no relevant data were calculated. The stratified analysis by sample size showed that according to the difference in sample size, the expression of miR-17-92 cluster miRNAs had no significant difference in AUC values for HCC detection (sample size < 100: AUC = 0.80; sample size > 100: AUC = 0.838). However, the miR-17-92 cluster miRNAs had higher sensitivity in the sample size < 100 group [sample size < 100: SEN = 0.79 (0.72–0.85); sample size > 100: SEN =0.69 (0.63–0.74)]. In addition, differences in sensitivity, specificity, and AUC were investigated by ethnicity. The results indicated that the sensitivity and AUC of miR-17-92 cluster miRNAs for HCC detection in the Chinese group were higher than those in the Egyptian group due to higher sensitivity and higher AUC [SEN=0.83 (0.78-0.88); AUC = 0.83]. Finally, the results of subgroup analysis of the tumor stage showed that the miR-17-92 cluster miRNAs had higher AUC and sensitivity in patients in the I–IV stage [SEN = 0.86 (0.78–0.92); AUC = 0.88].

**TABLE 2:** Subgroup analysis of the diagnostic value of the miR-17-92 cluster in HCC.

Subgroup		Pooled results						
		AUC	SEN (95% CI)	*I* ^2^ (%)	*p*	SPE (95% CI)	*I* ^2^ (%)	*p*
Sample type	Serum	0.831	0.69 (0.64–0.73)	86.9	0.000	0.78 (0.72–0.82)	18.8	0.2961
	Plasma	/	/	/	/	/	/	/
Sample size	<100	0.80	0.79 (0.72–0.85)	57.2%	0.9167	0.66 (0.59–0.72)	62.8	0.0681
	>100	0.838	0.69 (0.63–0.74)	91.3	0.0000	0.78 (0.72–0.83)	45.9	0.1575
Country	China	0.839	0.83 (0.78–0.88)	41.3	0.1821	0.66 (0.59–0.72)	61.6	0.0767
	Egypt	0.727	0.62 (0.56–0.68)	0.0	0.5279	0.78 (0.72–0.84)	41.5	0.1811
Tumor stage	I–IV	0.881	0.86 (0.78–0.92)	0	1	0.75 (0.62–0.85)	0	1
	NR	0.786	0.69 (0.64–0.73)	79.8	0.0005	0.71 (0.67–0.76)	75.3	0.0027

AUC, the area under the curve; SEN, sensitivity; SPE, specificity; NR, not reported.

### The results of prognostic meta-analysis of hepatocellular carcinoma

In the 15 studies (Fan et al., 2013; Zheng et al., 2013; Hung et al., 2015; Su et al., 2015; Yang et al., 2015; Wang et al., 2018; Liu et al., 2020a; Yang et al., 2020) enrolled for ascertaining the relationship between the miR-17-92 cluster and the prognosis of HCC patients, a total of 719 participants were identified for OS/DFS/RFS/PFS in this meta-analysis. The characteristics and methods of the included studies related to prognosis are shown in Table 3. Eligible included studies in the prognostic analysis were all from one country. In addition, seven of the included studies were based on the *in situ* hybridization (ISH) detection method, and the remaining studies were based on the qRT-PCR detection method.

**TABLE 3:** Main characteristics of the eligible studies for prognostic meta-analysis.

First author	Year	Country	Patient	Sample type	Sample stage	microRNA	Test method	Cut-off	Outcome	HR (95%CI)	NOS score
Beng Yang	2020	China	42	Blood	I–IV	miR-92a	ISH	NR	DFS	2.13 (1.53–8.53)	6
Chung-Lin Hung	2015	China	81	Fresh tissues	II–IV	miR-19b	qRT-PCR	NR	DFS	0.453 (0.245–0.845)	6
Jianjian Zheng	2013	China	96	Blood	I–IV	miR-17-5p	qRT-PCR	Median	OS	2.192 (1.024–4.691)	8
Beng Yang	2020	China	42	Blood	I–IV	miR-92a	ISH	NR	OS	2.7 (1.44–8.49)	6
Chung-Lin Hung	2015	China	81	Fresh tissues	II–IV	miR-19b	qRT-PCR	NR	OS	0.318 (0.12–0.846)	6
Dong-Li Liu	2020	China	104	Fresh tissues	I–IV	miR-17-5P	ISH	Mean	OS	0.7 (0.27–1.84)	7
Dong-Li Liu	2020	China	104	Fresh tissues	I–IV	miR-20a	ISH	Mean	OS	0.78 (0.19–1.05)	7
Ming-Qi Fan	2013	China	100	FFPE	I–III	miR-20a	qRT-PCR	NR	OS	4.937 (2.221–9.503)	6
Wei Yang	2015	China	106	Fresh tissues	I–IV	miR-92a	qRT-PCR	Median	OS	2.283 (1.104–4.717)	8
Xiaodong Wang	2018	China	123	Fresh tissues	I–IV	miR-18a	qRT-PCR	Mean	OS	6.29 (3.12–12.68)	7
Xiaoping Su	2015	China	90	FFPE	II–IV	miR-92a	ISH	NR	OS	2.49 (1.37–4.51)	6
Dong-Li Liu	2020	China	104	Fresh tissues	I–IV	miR-17-5P	ISH	Mean	PFS	0.4 (0.19–0.85)	7
Dong-Li Liu	2020	China	104	Fresh tissues	I–IV	miR-20a	ISH	Mean	PFS	0.46 (0.21–0.99)	7
Ming-Qi Fan	2013	China	100	FFPE	I–III	miR-20a	qRT-PCR	NR	RFS	4.281 (3.316–6.741)	6
Wei Yang	2015	China	106	Fresh tissues	I–IV	miR-92a	qRT-PCR	Median	RFS	3.706 (1.079–5.155)	8

NR, not reported; FFPE, formalin-fixed and paraffin-embedded; ISH, *In Situ* Hybridization; qRT-PCR, quantitative reverse transcription-polymerase chain reaction; OS, overall survival; DFS, disease-free survival; PFS, progression-free survival; RFS, recurrence or relapse-free survival; HR, hazard ratio; CI, confidence interval; NOS, Newcastle–Ottawa scale.

**FIGURE 9:**
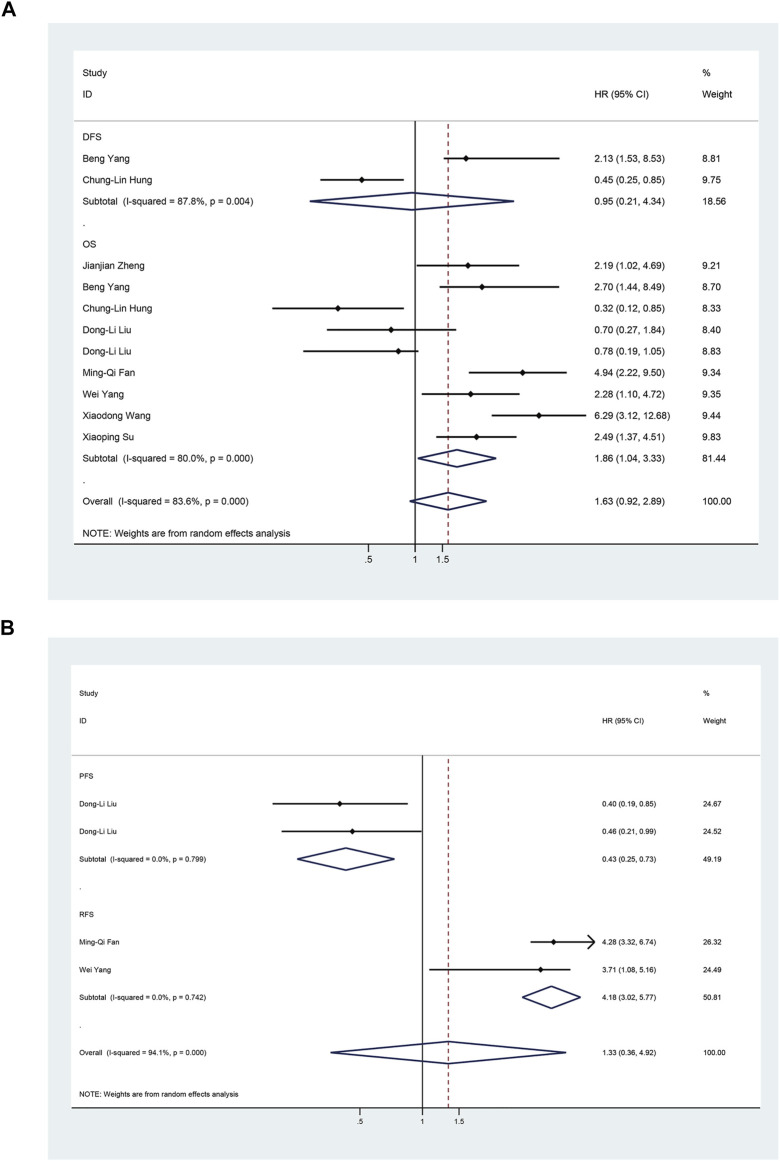
Forest plot of studies evaluating the hazard ratio of high miR-17–92 cluster expression in association with survival outcomes in hepatocellular carcinoma patients. **(A)** Studies are based on overall survival (OS) and disease-free survival (DFS); **(B)** Studies are based on Recurrence-Free Survival (RFS) and Progression-Free-Survival (PFS).

To assess the association between miR-17-92 cluster expression and OS, DFS, RFS, and PFS in HCC, the forest plot and meta-analysis of individual HR estimates are shown in Figure 9. The miR-17-92 cluster miRNA expression levels and the outcome of OS in HCC patients were examined in nine studies. In nine studies evaluating OS, the pooled HR and its 95% CI were calculated using a random-effects model with a result of 1.86 (95% CI: 1.04–3.33) because of a statistically significant heterogeneity (*I* ^2^ = 80.0 %, *p* = 0.000). Unexpectedly, there was also a significant heterogeneity between the two studies involving DFS (*I* ^*2*^ = 87.8%, *p* = 0.742), and the pooled result was HR = 0.95 (95% CI: 0.21–4.34). In addition, in enrolled studies appraising the RFS and PFS, the pooled HR and its 95% CI were 4.18 (3.02–5.77) and 0.43 (0.25–0.73), respectively, but no apparent heterogeneity was observed (*I* ^*2*^ = 0.0%, *p* = 0.742; *I* ^2^ = 0.0%, and *p* = 0.799, respectively). These results demonstrated that high expression of the miR-17-92 cluster miRNA is an unfavorable factor associated with OS and PFS, but a favorable factor for RFS. In fact, no correlation between the miR-17-92 cluster and DFS was detected.

The results of the heterogeneity test are shown in Table 4. Subgroup analyses were performed to find the sources that there was significant heterogeneity across studies for the pooled analysis of the miR-17-92 cluster and OS in the HCC patients. Subgroup analysis of sample types found that high expression of miR-17-92 cluster miRNAs in blood and FFPE samples was significantly associated with poor OS (HR = 2.39, 95 CI%: 1.34–4.27, *I* ^2^ = 0.00%, *P*_Heteqrogennity_ = 0.727; HR = 3.39, 95 CI%: 1.74–6.61, *I* ^*2*^ = 50.9%, *P*_Heteqrogennity_ = 0.153). However, the miR-17-92 cluster miRNA expression was not associated with OS in fresh tissue samples (HR = 1.24, 95 CI%: 0.44–3.57, *I* ^2^ = 87.4%, *P*_Heteqrogennity_ = 0.000). Similarly, in the subgroup analysis of tumor stage, when the cases were stage I–IV, the high expression of miR-17-92 cluster miRNA was also unfavorable for prognosis of HCC patients, and the pooled HR and its 95% CI was 1.96 (1.02–3.78). Furthermore, no significant associations were found between high or low expression of the miR-17-92 cluster and OS in subgroups of sample size (sample size ≤100 and sample size >100) and test method (ISH or qRT-PCR).

**TABLE 4:** Results of quantitative analysis.

Subgroup	HR	95% CI	I^2^ (%)	*P*_H_
OS				
Overall	1.86	1.04–3.33	80.0	0.000
Sample type				
blood	2.39	1.34–4.27	0	0.727
FFPE	3.39	1.74–6.61	50.9	0.153
Fresh Tissues	1.24	0.44–3.53	87.4	0.000
Test method				
ISH	1.44	0.71–2.92	66.7	0.029
qRT-PCR	2.27	0.93–5.56	85.3	0.000
Sample size				
≤100	1.96	0.90–4.25	80.2	0.000
>100	1.73	0.62–4.82	84.7	0.000

HR, hazard ratio; OS, overall survival; DFS, disease-free survival; FFPE, formalin-fixed and paraffin-embedded; *P*_H_, p heterogeneity.

**FIGURE 10:**
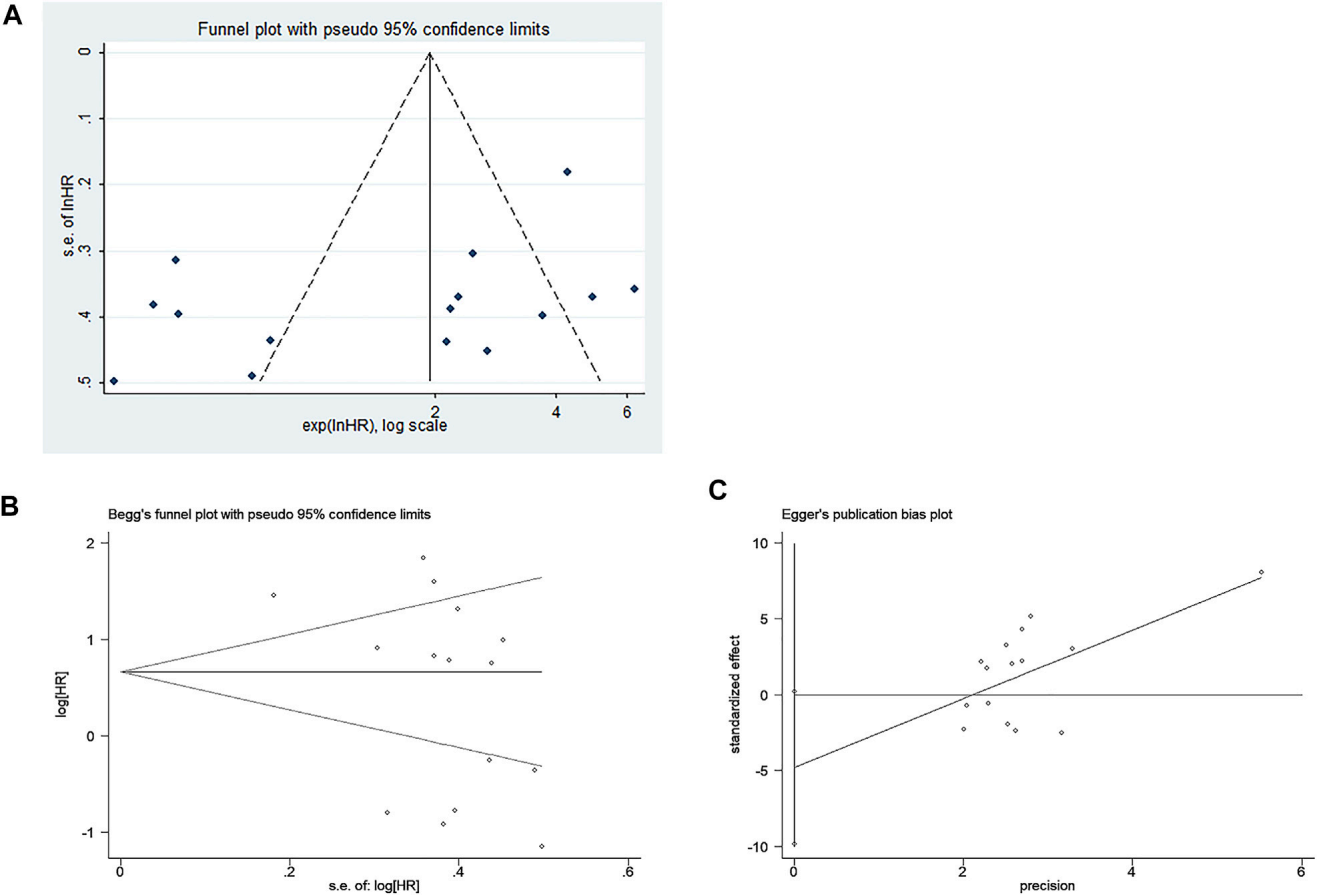
Publication bias of included studies for the prognostic meta-analysis. **(A)** Funnel plot; **(B)** Begg’s test; **(C)** Egger’s test.

**FIGURE 11:**
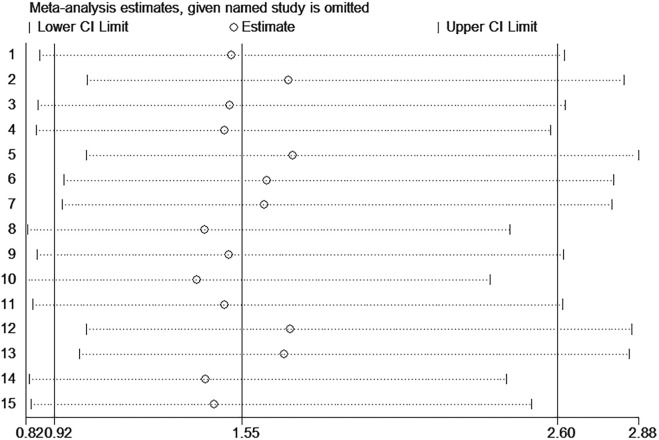
Sensitivity analysis of the miR-17–92 cluster for the prognosis of hepatocellular carcinoma patients.

The funnel plot was symmetrical, and Begg’s test (*p* = 0.166) and Egger’s test (*p* = 0.059) also indicated that there was no significant publication bias in these studies, as shown in Figure 10. Additionally, sensitivity analysis of OS, DFS, RFS, and PFS was performed, and every single study here was trimmed at a time to assess the specific effect of the single study on the pooled HRs, and the results suggested that pooled results were relatively stable, as shown in Figure 11.

## Discussion

In this meta-analysis, a total of 12 articles were included to determine the value of miR-17-92 cluster miRNAs in HCC diagnosis and prognosis. The pooled results showed that the expression of the miR-17-92 cluster could be used as a new diagnosis and prognosis biomarker for HCC.

In the present study, a total of six diagnosis-related studies were included to determine the association of abnormal miRNA expression levels of the miR-17-92 cluster with the diagnosis of HCC patients. In conclusion, the miR-17-92 cluster discriminated HCC from controls with an AUC value of 0.79 (0.76–0.83). For different diagnostic tests, the AUC value that can be calculated closer to one indicates a better test (Jones and Athanasiou, 2005). The results of the overall and subgroup analyses indicated that the miR-17-92 cluster had an acceptable moderate diagnostic value in HCC.

In fact, published studies have evidenced that the miR-17-92 cluster miRNAs have relatively high diagnostic value in HCC patients. For example, based on 11 GEO and TCGA databases, a meta-analysis showed (Chi et al., 2018) that the AUC of the sROC reached 0.88 (0.85–0.91), suggesting a certain distinguishing value of the miR-18a-5p in HCC, which was consistent to some extent with the results of the current study. In addition, previous studies have also demonstrated that the miR-17-92 cluster miRNAs can be used as a biomarker for HCC screening when used in combination with other miRNAs. For instance, Wen et al. (2015) reported that an eight-miRNA panel (miR-20a-5p, miR-25-3P, miR-30a-5p, miR-92a-3p, miR-132-3p, miR-185-5P, miR-320a, and miR-324-3P) had a diagnostic sensitivity and specificity of 86.6% and 64.6% for the HCC patients from the controls, respectively. In HCC following orthotopic liver transplantation (OLT), it had been reported that, compared with the miR-19a, the six-miRNA signature (miR-19a, miR-24, miR-126, miR-147, miR-223, and miR-886-5P) could improve the sensitivity and specificity from 71.9% and 71.4% to 86.7% and 82.3%, respectively (Han et al., 2012). Moreover, Sorop et al. (2020) reported that, together with serum AFP, plasma exosomal miR-21-5p and miR-92a-3p could be performed significantly better than AFP alone, whose AUC value for HCC diagnosis can increase from 0.72 to 0.85.

In recent years, accumulating evidence described the molecular mechanism of abnormal expression of miR-17-92 cluster miRNAs through several complex pathways in HCC. In the tumor microenvironment, exosomal miR-92a-5p from macrophages could inhibit AR translation and increase the invasive ability of hepatoma cells by altering the PHLPP/p-AKT/β-catenin signaling pathway (Liu et al., 2020b) Zhang et al. (2021) reported that overexpression of miR-20a could directly target EZH1, which was significantly upregulated in HCC and may be involved in H3K27 methylation, causing cell proliferation and gene transcriptional repression, ultimately leading to tumorigenesis. For the TGF-β signaling pathway, miR-17 bound to the three untranslated regions (3-UTR) of Smad3 mRNA and inhibited its protein expression at the posttranscriptional level (Lu et al., 2019).

In this study, the ROC plane showed an atypical shoulder-arm appearance with a Spearman correlation coefficient of −0.95 (*p* = 0.90), and there was no threshold effect on the heterogeneity. Therefore, our inclusion of diagnostic-related primary analysis results was relatively precise. Furthermore, it was meta-regression analysis that discovered such factors as different sample sizes of HCC patients and different sample types were probably important sources of heterogeneity. The reason might be that sample sources and sample size could affect the detection results of miRNA levels.

Studies have shown that a cluster of miRNAs may be a better predictor of survival than a single miRNA (Sapre et al., 2014). The role of miR-17-92 cluster miRNAs in various tumors has been demonstrated by statistical analysis (Zhang et al., 2017), which can serve as therapeutic prognostic biomarkers for various cancers. Indeed, the miR-17-92 cluster has been shown to play critical regulatory roles in the occurrence, metastasis, and prognosis of several cancer types. For example, miR-18a-5p inhibits tumor growth by targeting matrix metalloproteinase-3, and high levels of miR-18a are associated with better OS in cisplatin-resistant ovarian cancer patients (QuiÃ±ones-DÃaz et al., 2020). Zhang et al. (2020) reported that inhibition of SERTAD3-dependent miR-92a alleviated the growth, invasion, and migration of prostate cancer cells by regulating the expression of the key genes of the p53 pathway, including p38, p53, and p21. In addition, the ectopic expression of miR-19a-3p contributes to HCC metastasis and chemoresistance by modulating PTEN expression and the PTEN-dependent pathways (Jiang et al., 2018). In short, the miR-17-92 cluster, as a potential diagnostic and prognostic biomarker for tumor diseases, has played a significant role in understanding the mechanisms and clinical treatment of different cancers.

For prognostic-related meta-analysis, there was a significant correlation between high expression of the miR-17-92 cluster and poor OS and poor RFS in HCC patients. However, the correlation of increasing expression of miR-17-92 cluster miRNAs with DFS in HCC was not detected. Additionally, the current meta-analysis detected a favorable correlation between high expression of the miR-17-92 cluster miRNA and PFS in HCC. A possible problem was that due to the relatively small number of included studies related to RFS/PFS/DFS, these results remain inconclusive and further research is needed to confirm them.

Since significant heterogeneity was presented in this prognostic meta-analysis, it was necessary to elucidate the factors influencing heterogeneity. The detection and quantification method, patient-related sample size (sample size ≤100 and sample size >100), and sample type were considered key factors that may affect the magnitude of heterogeneity. Subgroup analysis showed that when samples were taken from blood and FFPE, high expression of the miR-17-92 cluster was associated with poorer OS in HCC; when the cases were stage I–IV, high expression of miR-17-92 cluster miRNA was significantly associated with poor OS in HCC patients. Therefore, when the samples were collected from blood or FFPE, the abnormal expression of miR-17-92 cluster miRNAs could indicate the OS of HCC patients. In short, there was a clear correlation between the miR-17-92 cluster and the prognosis of HCC patients, which can serve as a new prognostic biomarker for HCC.

Ultimately, certain limitations were presented in our meta-analysis: 1) none of the studies ultimately included in this study had both diagnostic and prognostic values; 2) the number of studies available for meta-analysis was still small, and the kind of ethnicity was monotonous; 3) among the included studies, the cut-off values were different or not reported; 4) the abnormal expression miR-17-92 cluster was previously reported for other human tumors, such as gastric and colorectal cancer. This phenomenon suggested that the high expression of miR-17-92 miRNA may be indirectly associated with HCC itself. Therefore, it will improve test performance when miR-17-92 cluster miRNAs are used in combination with other biomarkers; 5) chemosensitivity of patients with HCC may also be a factor affecting the prognosis, which is related to the abnormal expression of miR-17-92 miRNAs in the patient's tissues or blood; and 6) studies that provided survival curves artificially provided relevant HRs and CIs through relevant software, which may cause a certain degree of bias in our analysis results. Larger sample size and deeper data analysis are required to validate our findings.

To the best of our knowledge, this study was the first meta-analysis to assess the potential roles of the miR-17-92 cluster for HCC. The miR-17-92 cluster is a promising biomarker for diagnosis and prognosis of HCC. The current results echo those of previously published studies reporting that targeted overexpression of the miR-17-92 cluster enhanced hepatocarcinoma-induced liver tumorigenesis (Zhu et al., 2015). Although the reasons for the inconsistent results of the included studies have not been systematically addressed, further studies are clearly needed to demonstrate that mature miRNAs of members of the miR-17-92 cluster may differ as prognostic biomarkers in HCC patients. Finally, this study did not assess the prognostic value of a combination of the miR-17-92 cluster and other miRNA markers. Therefore, larger-size, multi-center, and higher-quality studies with a unified criterion for determining the expression of miR-17-92 cluster miRNAs are required.

## Data Availability

The original contributions presented in the study are included in the article/Supplementary Material; further inquiries can be directed to the corresponding authors.
